# Adapted systemic inflammation score as a novel prognostic marker for esophageal squamous cell carcinoma patients

**DOI:** 10.1002/ags3.12464

**Published:** 2021-06-15

**Authors:** Daichi Nomoto, Yoshifumi Baba, Takahiko Akiyama, Kazuo Okadome, Masaaki Iwatsuki, Shiro Iwagami, Yuji Miyamoto, Naoya Yoshida, Masayuki Watanabe, Hideo Baba

**Affiliations:** ^1^ Department of Gastroenterological Surgery Graduate School of Medical Sciences Kumamoto University Kumamoto Japan; ^2^ Department of Next‐Generation Surgical Therapy Development Graduate School of Medical Sciences Kumamoto University Kumamoto Japan; ^3^ Department of Gastroenterological Surgery Cancer Institute Hospital Japanese Foundation for Cancer Research Tokyo Japan; ^4^ Center for Metabolic Regulation of Healthy Aging Kumamoto University Kumamoto Japan

**Keywords:** adapted systemic inflammation score, esophageal squamous cell carcinoma, prognosis

## Abstract

**Background:**

The adapted systemic inflammation score (aSIS), calculated from serum albumin and the lymphocyte‐to‐monocyte ratio, has been reported to be a novel prognostic marker for some types of cancers. However, the prognostic impact of aSIS in patients with esophageal squamous cell carcinoma (ESCC) remains controversial. This study aimed to examine the prognostic effects of aSIS in a large cohort of 509 ESCC patients.

**Methods:**

Preoperative aSIS was retrospectively calculated for 509 ESCC patients who underwent curative resection. Time‐dependent receiver operating characteristics (t‐ROC) curves were used for comparing the prognostic impact.

**Results:**

Patients with high aSIS showed significantly poorer overall survival (OS) than patients with low aSIS (log rank *P* < .001). The multivariate analysis revealed that aSIS was an independent prognostic factor for overall survival (multivariate hazard ratio 1.76; 95% confidence interval 1.13–2.75; *P* = .013). The t‐ROC analysis showed that aSIS was more sensitive than other nutritional prognostic factors (controlling for nutritional status, systemic inflammation score, and the neutrophil‐to‐lymphocyte ratio).

**Conclusion:**

Preoperative aSIS may be a useful prognostic biomarker in ESCC patients who underwent curative resection.

## Graphical abstract

This study examined the prognostic effects of the adapted systemic inflammation score (aSIS), calculated from serum albumin and the lymphocyte‐to‐monocyte ratio, in a large cohort of 509 esophageal squamous cell carcinoma (ESCC) patients. ESCC patients with high aSIS showed significantly poorer overall survival than patients with low aSIS. aSIS was more sensitive than other nutritional prognostic factors (controlling for nutritional status, systemic inflammation score, and the neutrophil‐to‐lymphocyte ratio).

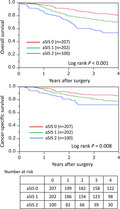

## 1 INTRODUCTION

Esophageal cancer is the sixth leading cause of cancer death and the seventh most common cancer worldwide.^1^ Despite the development of multidisciplinary treatment strategies, including surgery, chemotherapy, radiotherapy, and chemoradiotherapy, the prognosis of patients with esophageal cancer, including those who underwent complete resection, remains poor. Thus, better prognostic biomarkers are needed for making appropriate treatment decisions for esophageal cancer patients.

The systemic inflammation score (SIS) was developed as a new scoring system based on serum albumin (Alb) and the lymphocyte‐to‐monocyte ratio (LMR). SIS has been reported as a powerful prognostic marker for clear‐cell renal cell carcinoma and colorectal cancer.^2^, ^3^ The cutoff value of LMR in SIS was the median value for patients with renal cell carcinoma. Therefore, Lin et al developed a modified SIS (mSIS) with optimized LMR cutoff values for gastric cancer patients using the software X‐tile (Yale University, New Haven, CT).^4^ The mSIS has been shown to be superior to SIS as a predictive indicator for gastric cancer.^5^ Several studies have examined the prognostic role of SIS and mSIS in esophageal squamous cell carcinoma (ESCC).^6^, ^7^, ^8^ However, previous data on SIS, mSIS, and clinical outcomes for ESCC have been inconclusive because previous studies were limited by small sample sizes (n < 443). Considering the importance of a potential prognostic marker for ESCC, the assessment of SIS and clinical outcome using a large number of ESCC cases is needed.

In this study we reoptimized the LMR cutoff values as 3.3 using the software X‐tile according to esophageal cancer patients’ cohort, and defined adapted SIS (aSIS). The prognostic effects of aSIS were examined in a large cohort of 509 ESCC patients. Our nonbiased database enabled us to examine whether the influence of aSIS on clinical outcome was modified by numerous other variables. Furthermore, the predictive value of aSIS was compared with that of other known nutritional prognostic factors (Controlling Nutritional Status [CONUT] and Neutrophil‐to‐Lymphocyte ratio [NLR]).

## 2 MATERIALS AND METHODS

### 2.1 Patients

Between April 2005 and December 2017, 751 consecutive patients with esophageal cancer who underwent curative resection at Kumamoto University Hospital were enrolled. Of these patients, patients who underwent salvage esophagectomy (53), pharyngolaryngoesophagectomy (27), transhiatal esophagectomy (11), and noncurative surgery (R1 or R2 resection) (53) and with other histological types (93) and who lack peripheral monocyte data (5) were excluded from the study. Thus, a total of 509 patients with esophageal cancer who underwent subtotal esophagectomy were eventually included in the study. We classified the clinical tumor stage according to the Union for International Cancer Control (UICC) TNM classification of malignant tumors, seventh edition.^9^ Patients were followed up as outpatients every 1–3 mo after discharge until death or December 2019, whichever came first. Overall survival (OS) was defined as the period from the date of surgery to the date of death. Cancer‐specific survival (CSS) was defined as the period from the date of surgery to the date of death owing to esophageal cancer. Patients' clinicopathological characteristics were obtained retrospectively from the medical records. These characteristics included age, sex, body mass index (BMI), comorbidity, preoperative therapy, clinical tumor stage, OS, and CSS. Written informed consent was obtained from each patient, and the study was approved by the Institutional Review Board of Kumamoto University (#1909). The term “prognostic marker” was used throughout this article according to the Reporting Recommendations for Tumor Marker Prognostic Studies (REMARK) guidelines.^10^

### 2.2 The aSIS and other scoring systems

**FIGURE 1:**
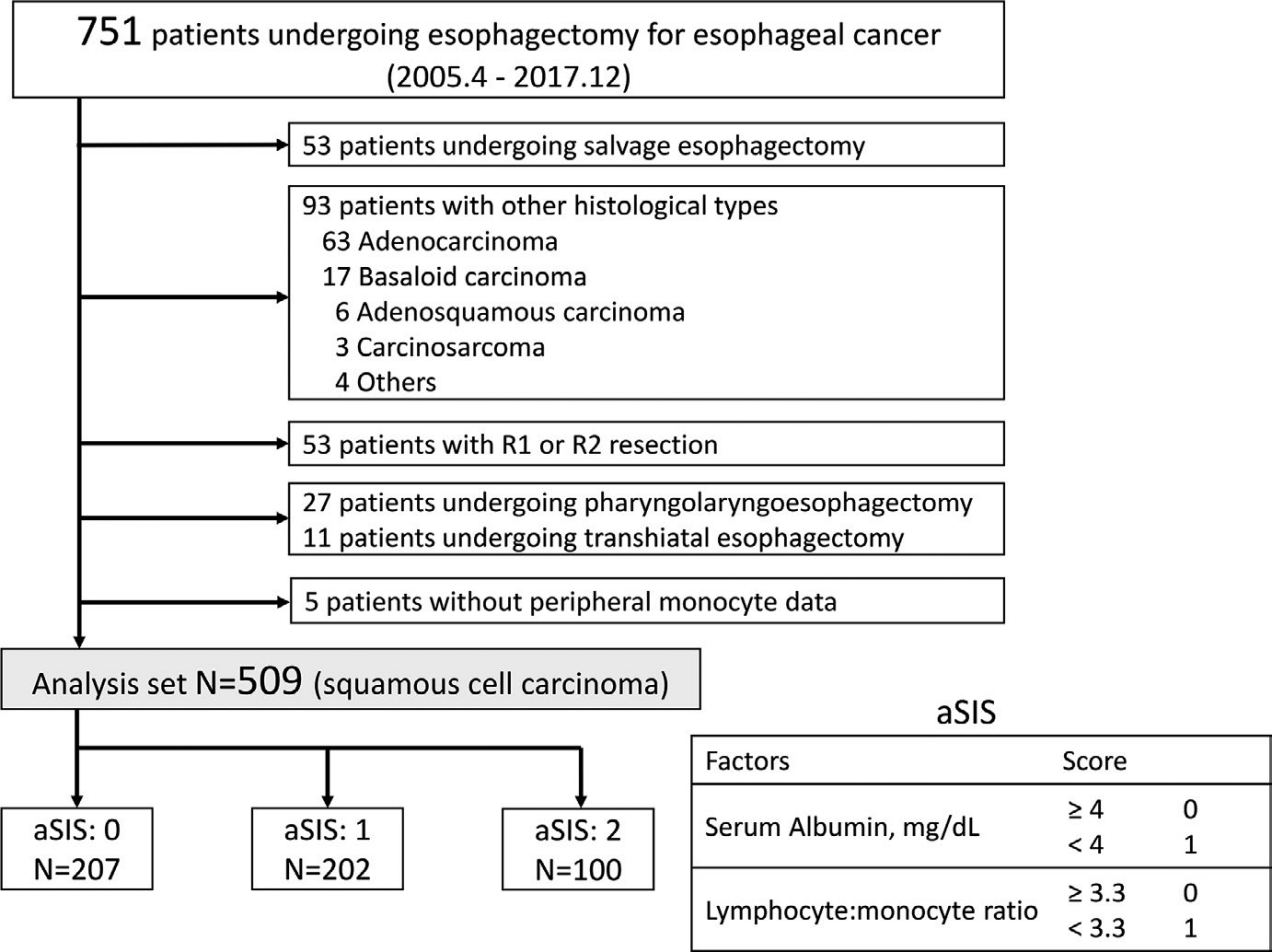
Flow chart of the analyzed cases and the definition of adapted systemic inflammation score

Serum samples were obtained and analyzed within 7 d before esophagectomy. The aSIS was calculated from serum albumin and peripheral LMR (Figure 1). The LMR cutoff value was calculated using the software X‐tile,^4^ which determined the LMR cutoff value as 3.3 (Figure S1).

NLR and CONUT scores were calculated as described previously.^11^, ^12^, ^13^

### 2.3 Statistical analyses

All statistical analyses were performed using JMP v. 10 (SAS Institute, Cary, NC) and EZR (Saitama Medical Center, Jichi Medical University, Japan). Categorical variables were presented as numbers or percentages, and the groups were compared using the chi‐squared test or Fisher's exact test. Continuous variables are presented as mean values and standard deviations, and the mean values were compared using Student's *t*‐test. For survival analysis, the Kaplan–Meier method was used for evaluating the survival time distribution, and the log‐rank test was performed for comparisons. The multivariate Cox proportional hazards model was constructed for computing hazard ratios (HRs) and 95% confidence intervals (CIs) according to aSIS containing clinicopathological factors potentially related to the clinical outcome. Time‐dependent receiver operating characteristic curves (ROCs) for the prognostic values of NLR, CONUT, SIS, and aSIS were estimated using the R package “timeROC” (https://cran.r‐project.org/web/packages/timeROC/index.html). Variables with *P* < .05 in the univariate analysis were subjected to the multivariate model using a stepwise backward elimination procedure with a threshold *P* < .05. Statistical significance was set at *P* < .05.

## 3 RESULTS

### 3.1 mSIS and clinicopathological characteristics

Of the 509 ESCC patients, 446 (88%) were men and 63 (12%) were women. The mean age was 66.3 y (range, 41–89 y). Patients were segregated into three groups based on the aSIS: 207 patients (40%) in the aSIS = 0 group, 202 (40%) in the aSIS = 1 group, and 100 (20%) in the aSIS = 2 group (Figure 1). The relationships between aSIS and clinicopathological factors are presented in Table 1. Older age, poorer performance status (PS), lower BMI, and higher clinical stage were significantly associated with higher aSIS (each *P* < .05).

**TABLE 1:** Patient characteristics

Variables	Total N	aSIS			*P* value
		0	1	2	
All cases	509	207	202	100	
Mean age ±SD	66.3 ± 8.0	64.6 ± 8.0	67.8 ± 7.8	67.2 ± 8.3	< .01
Sex, male	446 (88%)	178 (86%)	176 (87%)	92 (92%)	.31
Performance status					
0	443 (87%)	190 (92%)	175 (87%)	78 (78%)	< .01
1	57 (11%)	17 (8%)	21 (10%)	19 (19%)	
2	9 (2%)	0 (0%)	6 (3%)	3 (3%)	
Body Mass Index					
<18.5	61 (12%)	16 (8%)	26 (13%)	19 (19%)	< .01
18.5 ≤, <25	368 (72%)	146 (70%)	151 (75%)	71 (71%)	
25≤	80 (16%)	45 (22%)	25 (12%)	10 (10%)	
Alcohol use, Yes	483 (95%)	197 (95%)	192 (95%)	94 (94%)	.91
Tobacco use, Yes	436 (86%)	168 (82%)	176 (87%)	92 (92%)	.026
Comorbidity, Present	392 (77%)	145 (70%)	166 (82%)	81 (81%)	< .01
Preoperative treatment					
Present	195 (38%)	44 (21%)	91 (45%)	60 (60%)	< .01
Absent	314 (62%)	163 (79%)	111 (55%)	40 (40%)	
Tumor location					
Ce	2 (1%)	1 (1%)	1 (1%)	0 (0%)	.71
Ut	72 (14%)	31 (15%)	32 (16%)	9 (9%)	
Mt	279 (55%)	112 (54%)	113 (56%)	54 (54%)	
Lt	143 (28%)	58 (28%)	51 (25%)	34 (34%)	
Ae	13 (2%)	5 (2%)	5 (2%)	3 (3%)	
Clinical stage					
I	252 (50%)	140 (68%)	89 (44%)	23 (23%)	< .01
II	89 (17%)	30 (14%)	39 (19%)	20 (20%)	
III	143 (28%)	26 (13%)	68 (34%)	49 (49%)	
IV	25 (5%)	11 (5%)	6 (3%)	8 (8%)	
Postoperative treatment					
Present	96 (19%)	50 (24%)	30 (15%)	16 (16%)	.053
Absent	413 (81%)	157 (76%)	172 (85%)	84 (84%)	

Abbreviations: aSIS, adapted systemic inflammation score; SD; standard deviation.

### 3.2 Correlations between the aSIS and short‐term outcomes of surgery

The short‐term outcomes after surgery according to the aSIS are presented in Table 2. High aSIS was significantly associated with a higher incidence of pulmonary morbidity (*P* < .01). Other complications and complication severity were not associated with aSIS. We checked the correlation between aSIS and patients' characteristics affecting pulmonary morbidity such as a respiratory function or surgical procedure. Regarding a respiratory function, we analyzed percent vital capacity (%VC) and forced expiratory volume in 1 sec as a percentage of forced vital capacity (FEV1.0%). %VC decreased significantly as aSIS increased (Figure S2; *P* < .001). On the other hand, there was no significant correlation between FEV1.0% and aSIS (Figure S2; *P* = .698). Regarding a surgical procedure, we checked minimally invasive esophagectomy (MIE) and open esophagectomy (OE). There were more patients with aSIS:2 and fewer patients with aSIS:0 in the OE group than in the MIE group (Figure S2; *P* = .011).

**TABLE 2:** Short‐term outcome of surgery

Variables	Total N	aSIS			*P* value
		0	1	2	
All cases (Subtotal esophagectomy)	509	207	202	100	
Operating time (min) ±SD	562 ± 121	579 ± 129	552 ± 115	548 ± 114	.033
Blood loss (g) ±SD	475 ± 556	467 ± 684	437 ± 382	568 ± 555	.15
Postoperative morbidity, CDc ≥II	193 (38%)	81 (39%)	67 (33%)	45 (45%)	.12
Postoperative morbidity, CDc ≥IV	33 (6%)	12 (6%)	11 (5%)	10 (10%)	.31
Surgical site infection	125 (25%)	56 (27%)	48 (24%)	21 (21%)	.48
Pulmonary morbidity	87 (17%)	31 (15%)	28 (14%)	28 (28%)	< .01
Cardiovascular morbidity	29 (6%)	12 (6%)	11 (5%)	6 (6%)	.98
Anastomotic leakage	64 (13%)	31 (15%)	18 (9%)	15 (15%)	.12
Reoperation	35 (7%)	14 (7%)	16 (8%)	5 (5%)	.63

Abbreviations: aSIS, adapted systemic inflammation score; CDc, Clavien–Dindo classification; SD, standard deviation.

### 3.3 Adapted systemic inflammation score and patient survival

**FIGURE 2:**
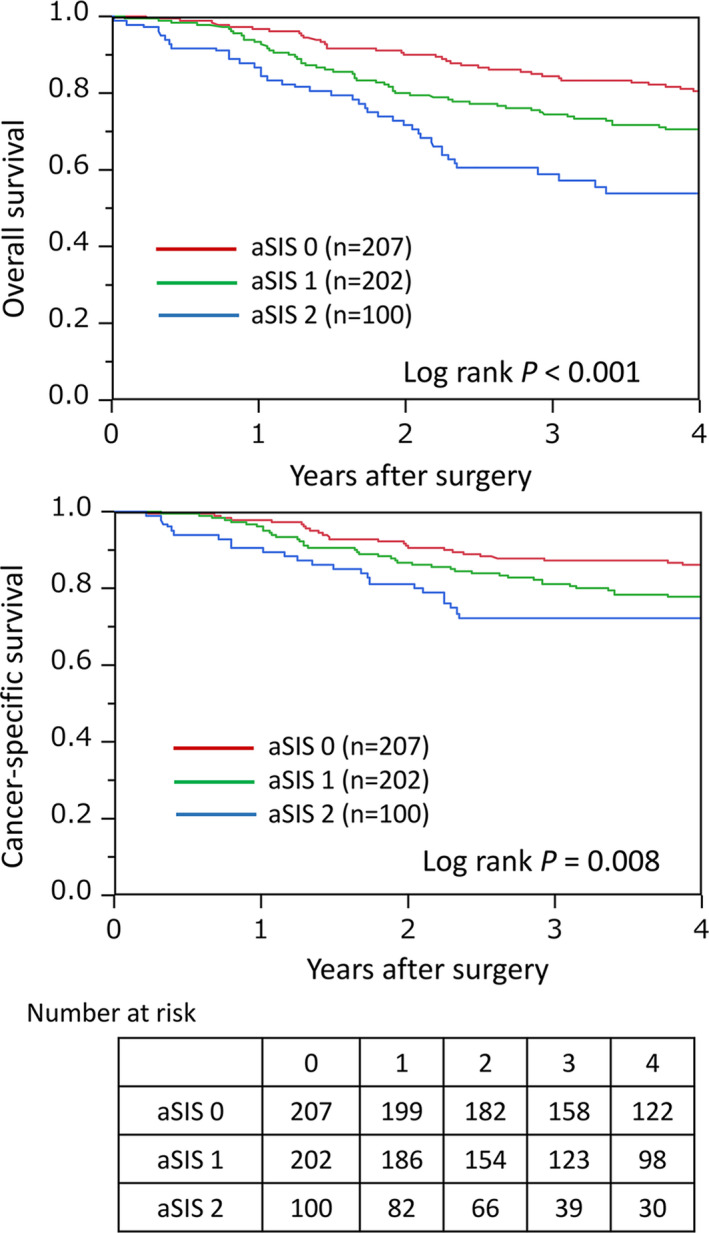
Kaplan–Meier curves for the overall survival (OS) and cancer‐specific survival in esophageal squamous cell carcinoma patients according to adapted systemic inflammation score stratification

The impact of aSIS on clinical outcomes was assessed in ESCC patients. There were 168 deaths among the 509 patients, including 96 esophageal cancer‐specific deaths. The median follow‐up time for censored patients was 4.0 y. The Kaplan–Meier analysis showed that there was a significant difference in the OS and CSS among the three groups (both log rank *P* < .01). The 3‐y OS rates in groups 0, 1, and 2 were 84.1%, 74.6%, and 57.3%, respectively (Figure 2). The 3‐y CSS rates in groups 0, 1, and 2 were 87.5%, 81.3%, and 72.0%, respectively (Figure 2).

In the univariate analysis, a high aSIS score was associated with poor OS (HR 2.50, 95% CI 1.65–3.79, *P* < .01) (Table 3). For patient factors, advanced age (HR 1.56, 95% CI 1.14–2.17, *P* < .01), lower BMI (HR 1.81, 95% CI 1.16–2.71, *P* < .01), and poor PS (HR 2.09, 95% CI 1.41–3.01, *P* < .01) were significantly associated with poor OS. For tumor factors, clinical stage (HR 2.19, 95% CI 1.60–2.97, *P* < .01) was significantly associated with poor OS. In the multivariate analysis, aSIS was an independent prognostic factor for the OS (multivariate HR 1.81, 95% CI 1.16–2.81, *P* < .01). The other independent prognostic factors were advanced age, low BMI, poor PS, and postoperative complications.

**TABLE 3:** Cox regression analysis for overall survival

Variables	Univariate analysis		Multivariate analysis	
	HR (95% CI)	*P* value	HR (95% CI)	*P* value
Age (≥ 65 vs <65)	1.56 (1.14–2.17)	< .01	1.46 (1.03‐2.07)	.032
Sex (male vs female)	1.18 (0.74–1.98)	.52		
Brinkman Index (≥800 vs <800)	1.05 (0.77–1.42)	.77		
Body Mass Index				
(≥25 vs <25)	0.72 (0.44–1.12)	.15		
(<18.5 vs ≥18.5)	1.81 (1.16–2.71)	< .01	1.67 (1.06–2.53)	.028
Performance Status (1, 2 vs 0)	2.09 (1.41–3.01)	< .01	1.54 (1.01–2.27)	.043
Comorbidity (+ vs –)	1.52 (1.04–2.29)	.028	1.16 (0.77–1.78)	.48
cStage (III, IV vs I, II)	2.19 (1.60–2.97)	< .01	1.38 (0.90–2.12)	.14
Preoperative treatment (+ vs −)	2.18 (1.59–2.97)	< .01	1.50 (0.98–2.27)	.060
Preoperative aSIS				
(1 vs 0)	1.76 (1.23–2.54)	< .01	1.36 (0.93–2.01)	.11
(2 vs 0)	2.50 (1.65–3.79)	< .01	1.81 (1.16–2.81)	< .01
Postoperative complications				
CDc ≥IIIb (+ vs −)	1.72 (1.11–2.56)	.016	1.74 (1.12–2.63)	.016

Abbreviations: CDc, Clavien–Dindo classification; CI, confidence interval; HR, hazard ratio.

### 3.4 Survival analysis of interactions between aSIS and other factors

**FIGURE 3:**
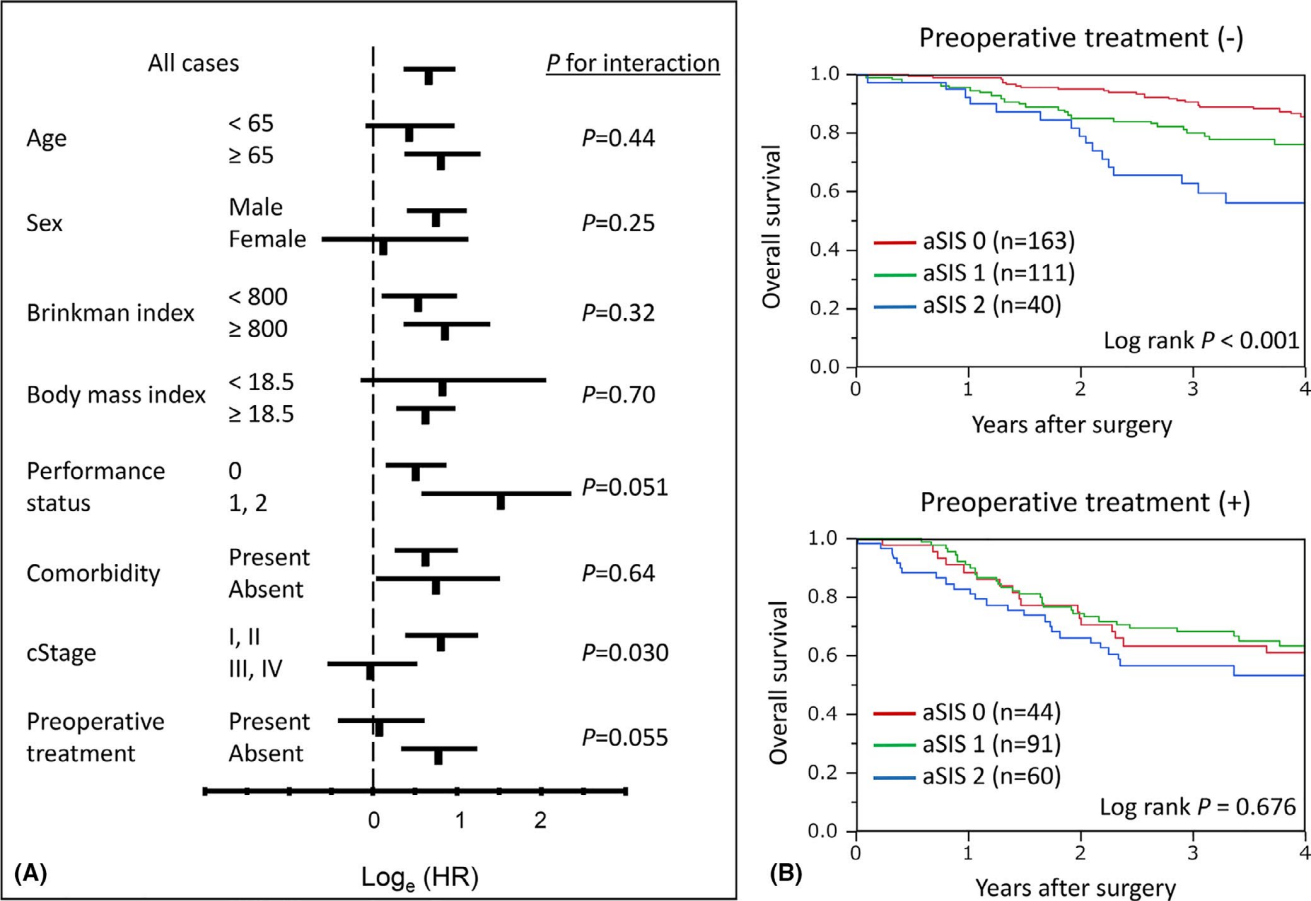
A: Relationship between the adapted systemic inflammation score (aSIS) and OS. Log_e_(HRs) plots of the OS rate in aSIS 0 (low) and aSIS 1, 2 (high) groups are shown. B: Kaplan–Meier curves for OS in patients with esophageal squamous cell carcinoma, according to the aSIS stratification. The upper panel includes patients who did not receive any preoperative treatment. The lower panel includes patients who received preoperative treatment. aSIS, modified systemic inflammation score; OS, overall survival; HRs, hazards ratio

We next determined whether the influence of aSIS on the OS was affected by any clinicopathological factors. The effect of aSIS was not significantly modified by comorbidity, BMI, Brinkman Index, sex, or age (*P* > .11 for all interactions) (Figure 3A). Interestingly, a potential modifying effect of preoperative treatment on the relationship between the aSIS and OS was observed (*P* for interaction = .055). Among patients who did not receive preoperative treatment, the high mSIS had a significantly shorter OS (log rank *P* < .001). In contrast, among patients who received preoperative treatment, there was no significant difference in the OS for the high aSIS and low aSIS (log rank *P* = .676) (Figure 3B). Similarly, a potential modifying effect of cStage on the relationship between the mSIS and OS was also observed (*P* for interaction = .030). Among patients with cStage I, II, the high mSIS had a significantly shorter OS (log rank *P* < .001) (Figure S3).

Furthermore, we divided the patients into those who received preoperative treatment and those who did not, and further analyzed the patients' characteristics and prognostic factors.

In patients without preoperative treatment, the relationships between aSIS and clinicopathological factors are presented in Table S1. Older age, poorer PS, lower BMI, and higher clinical stage were significantly associated with higher aSIS (each *P* < .05). High aSIS was significantly associated with a higher incidence of pulmonary morbidity (*P* < .01). In the multivariate Cox regression analysis for OS, aSIS was an independent prognostic factor for the OS (multivariate HR 2.27, 95% CI 1.18–4.19, *P* = .02; Table S3). These results for patients who did not receive preoperative treatment were in good agreement with the analysis results of all 509 patients.

On the other hand, in patients with preoperative treatment, the relationships between aSIS and clinicopathological factors are presented in Table S2. Older age, and smoker were significantly associated with higher aSIS (each *P* < .05). Unlike patients without preoperative treatment, high aSIS was not significantly associated with a higher incidence of pulmonary morbidity (*P* = .62). Furthermore, in the univariate and multivariate Cox regression analysis for OS, aSIS was not an independent prognostic factor for the OS (univariate HR 1.23, 95% CI 0.68–2.26, *P* = .50; Table S4).

### 3.5 Comparison of aSIS with other prognostic scoring systems

**FIGURE 4:**
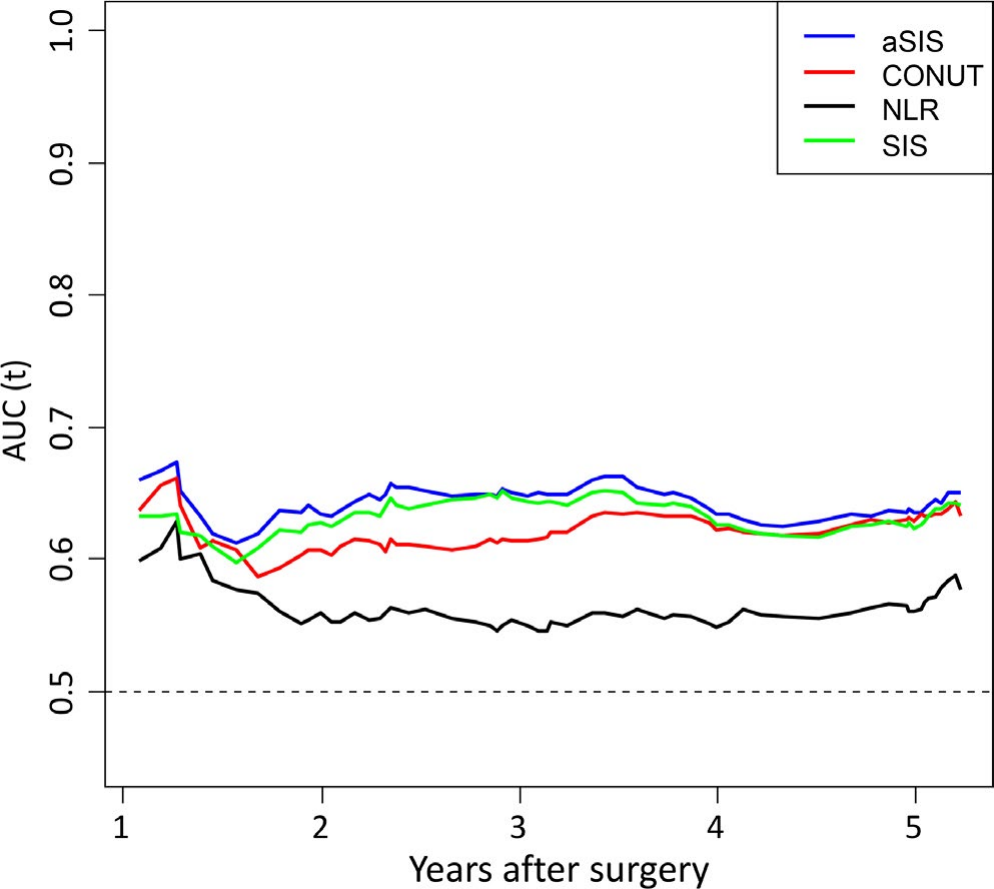
The time‐dependent AUC‐of‐ROC curves of aSIS and other prognostic scoring systems (CONUT, SIS, NLR) for the prediction of OS. AUC‐of‐ROC, area under the curve of the receiver operating characteristics; CONUT, controlling nutritional status; SIS, systemic inflammation score; aSIS, adapted systemic inflammation score; NLR, neutrophil‐to‐lymphocyte ratio

Time‐dependent area under the curve (AUC)‐of‐ROC curves of aSIS and other prognostic scoring systems (CONUT, SIS, and NLR) was constructed for the prediction of OS (Figure 4). When the predictive performance for the OS was compared after 1–5 y of follow‐up, the AUC values of the aSIS were similar to those of the SIS and CONUT, and were significantly higher than those of the NLR.

## 4 DISCUSSION

In this study we examined the prognostic effects of aSIS in a large cohort of 509 patients with ESCC. We found that high aSIS was associated with poor prognosis, suggesting that aSIS status could be used as a marker to identify patients who are likely to have unfavorable clinical outcomes. Additionally, the t‐ROC analysis showed that aSIS was more sensitive than other nutritional prognostic factors (CONUT, NLR, and SIS). Given that better prognostic biomarkers are needed for making appropriate treatment decisions for patients with esophageal cancer, our observations are likely to have clinical implications.

Systemic inflammation plays an important role in the tumor microenvironment. Several investigations have revealed that inflammation in the tumor microenvironment promotes tumor proliferation, angiogenesis, metastasis, inhibition of tumor immunity, and resistance to anticancer treatment.^14^ Several studies have also shown the predictive value of nutrition and systemic inflammation markers (eg, CONUT and NLR) in patients who underwent curative resection for various cancers. CONUT was calculated from the serum Alb level, total cholesterol concentration, and total lymphocyte count. NLR was calculated from the neutrophil‐to‐lymphocyte ratio.

SIS is a new scoring system based on serum Alb and LMR. Chang et al first reported that SIS predicts the postoperative prognosis of patients with clear‐cell renal cell carcinoma, and the cutoff value of LMR was 4.44 based on the median value of LMR.^2^ Higher SIS was associated with poorer OS in colorectal cancer^3^, ^15^ and gastric cancer.^16^, ^17^ However, Lin et al showed that SIS was not an independent prognostic factor for gastric cancer patients based on a multivariate analysis.^5^ They determined a new cutoff value of LMR at 3.4 using the software X‐tile^4^ and constructed the mSIS. The mSIS was the only significant independent biomarker, and a higher mSIS score was associated with 5‐y OS.

Two previous studies on ESCC showed that a high SIS was significantly associated with poorer OS.^6^, ^7^ There was only one study focusing on the prognostic role of mSIS in ESCC patients.^8^ Kanda et al analyzed patients with esophageal cancer using Lin's cutoff value of 3.4, without using the software X‐tile, and reported that a higher mSIS was associated with poorer disease‐free survival in 443 ESCC patients.^8^ However, all of these studies were limited by small sample sizes (n < 450). In contrast to previous studies, our study evaluated preoperative aSIS in a much larger cohort of over 500 ESCC patients. Furthermore, the LMR cutoff value was optimized at 3.3 for ESCC patients using the software X‐tile. In our study aSIS was a significant independent prognostic biomarker and the higher aSIS was associated with poorer OS and CSS. Importantly, aSIS was more sensitive than other nutritional prognostic factors, namely, CONUT, NLR, and SIS. The reason why aSIS was more sensitive than SIS might be the difference in the LMR cutoff value (the LMR cutoff values in aSIS and SIS were 3.3 and 4.44, respectively). In ESCC patients, The LMR cutoff value 4.44 was strict and many patients were assigned to SIS:2 (SIS 2:32%, aSIS 2:20%), and it might not be possible to stratify patients with poor SIS. The reason why aSIS was more sensitive than CONUT and NLR might be the difference in each prognostic factor's components. Especially, it might be affected by the fact that only aSIS includes monocytes, not CONUT and NLR. Peripheral monocyte count appeared to be useful prognostic marker in a large cohort of patients with ESCC.^18^ However, the precise molecular mechanism by which monocytes might predict clinical survival is not understood. One hypothesized pathogenic mechanism is that monocytes are recruited by some cytokines and chemokines around the tumor and differentiate into tumor‐associated macrophages (TAMs), which facilitate multiple tumorigenic mechanisms. Type 2 macrophages, in particular, have a tumorigenic effect that includes tumor initiation, invasion, angiogenesis, and metastasis.^19^ We have previously reported that the presence of a high‐density of TAMs was associated with significantly worse OS in patients with resected ESCC.^20^ Therefore, an elevated absolute monocyte count may indicate the presence of TAMs.

The biological mechanism underlying the relationship between the systemic impact of aSIS and prognosis is still unknown. One mechanism may involve the relationship between systemic inflammation and tumor immunity (tumor infiltrating lymphocytes [TILs]). We have previously revealed the correlation between TIL status and total lymphocyte count in peripheral blood. TILs might promote antitumor immunity and might be associated with improved outcomes in ESCC patients.^21^ Further experimental studies are necessary for investigating these mechanisms in ESCC.

Interestingly, we found that the prognostic effect of the aSIS differed according to the neoadjuvant treatment status. Low aSIS was associated with high OS among patients who did not undergo neoadjuvant treatment. In contrast, in patients who underwent neoadjuvant treatment, there was no association between the aSIS and OS. The most probable reason could be that many patients with neoadjuvant treatment have advanced‐stage ESCC and poor prognosis (Table S2). On the other hand, 50% of patients without neoadjuvant treatment were cStage I (Table S1). Second, in the clinical setting for esophageal cancer treatment, preoperative therapy might negatively affect the patients’ systemic nutritional and immunological status. In fact, comparing preneoadjuvant treatment and preoperative blood sampling values, Alb levels and lymphocyte count decreased significantly (Alb 4.0 ± 0.4 vs 3.7 ± 0.4, *P* < .01; lymphocytes 1766 ± 597 vs 1486 ± 584, *P* < .01), and monocyte count tended to decrease (434 ± 174 vs 413 ± 160, *P* = .14). Approximately 67% of the patients with aSIS of 0 before neoadjuvant treatment showed elevated preoperative aSIS. This could affect their clinical outcome via the local tumor immune response.^22^

This study has several limitations. First, this was a retrospective study. Second, the patients were included from just our institution. The significance of aSIS needs to be validated using other cohorts.

In conclusion, our large cohort study with more than 500 cases revealed that the preoperative aSIS was associated with the clinical outcome in ESCC patients who underwent curative resection. Furthermore, aSIS was more sensitive than other nutritional prognostic factors. Considering the relationship between nutritional status and local tumor immunity, our findings may have considerable clinical implications. Future studies are needed for confirming our findings as well as for elucidating the exact mechanisms by which the nutritional status affects local tumor immunity.

## CONFLICT OF INTEREST

No conflicts of interest exist.

## ETHICAL STATEMENT

The study was approved by the Institutional Review Board of Kumamoto University (#1909).

## Supporting information

Fig S1

Fig S2

Fig S3

Table S1‐4

Supplementary Material
