# Supplementary figures and images for: Adapted systemic inflammation score as a novel prognostic marker for esophageal squamous cell carcinoma patients
Source: Ann Gastroenterol Surg. 2021 Jun 15;5(5):669–76. doi: 10.1002/ags3.12464 (PMC8452479; doi:10.1002/ags3.12464)

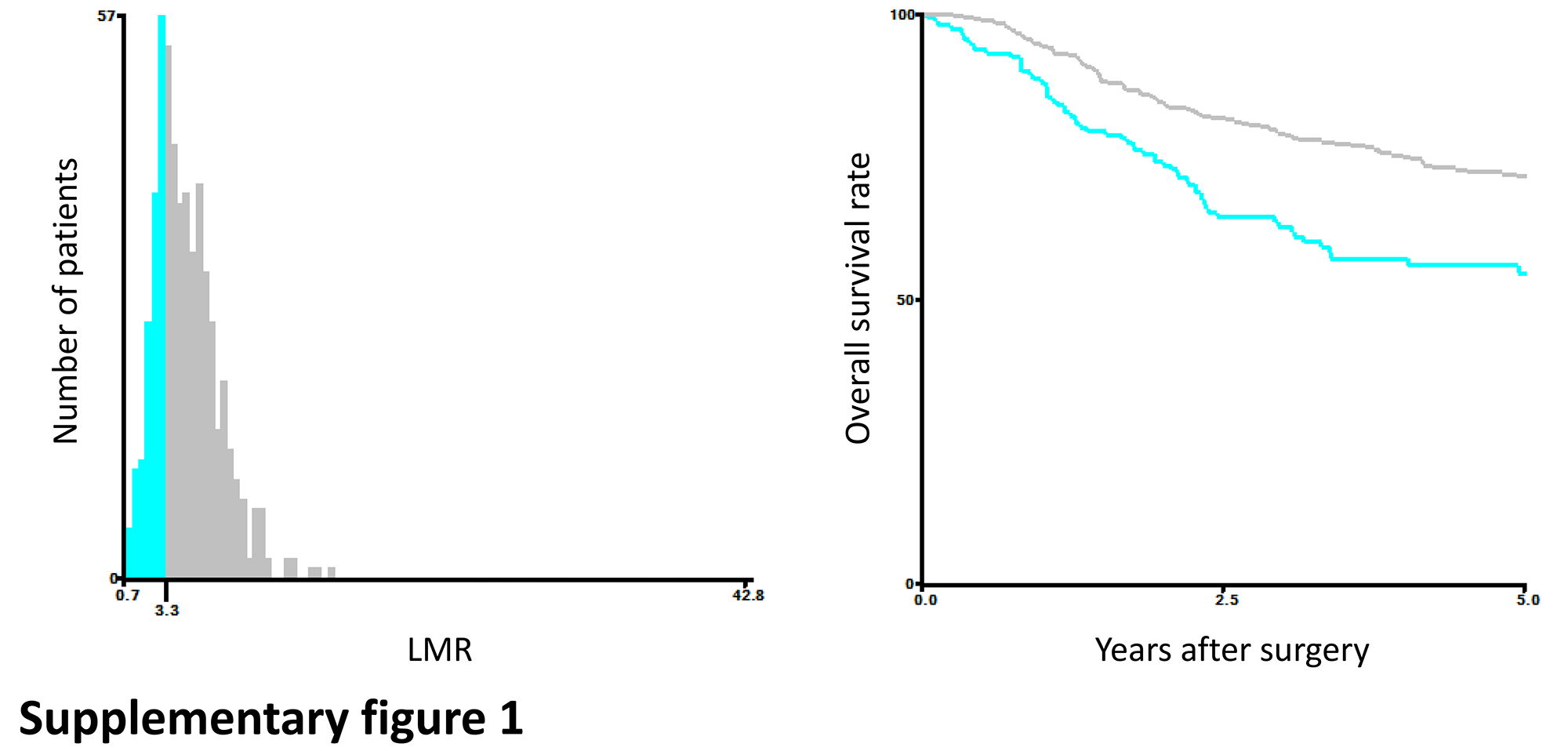

Supplement: Supplementary file 1 — Fig S1 [file AGS3-5-669-s003.tif]

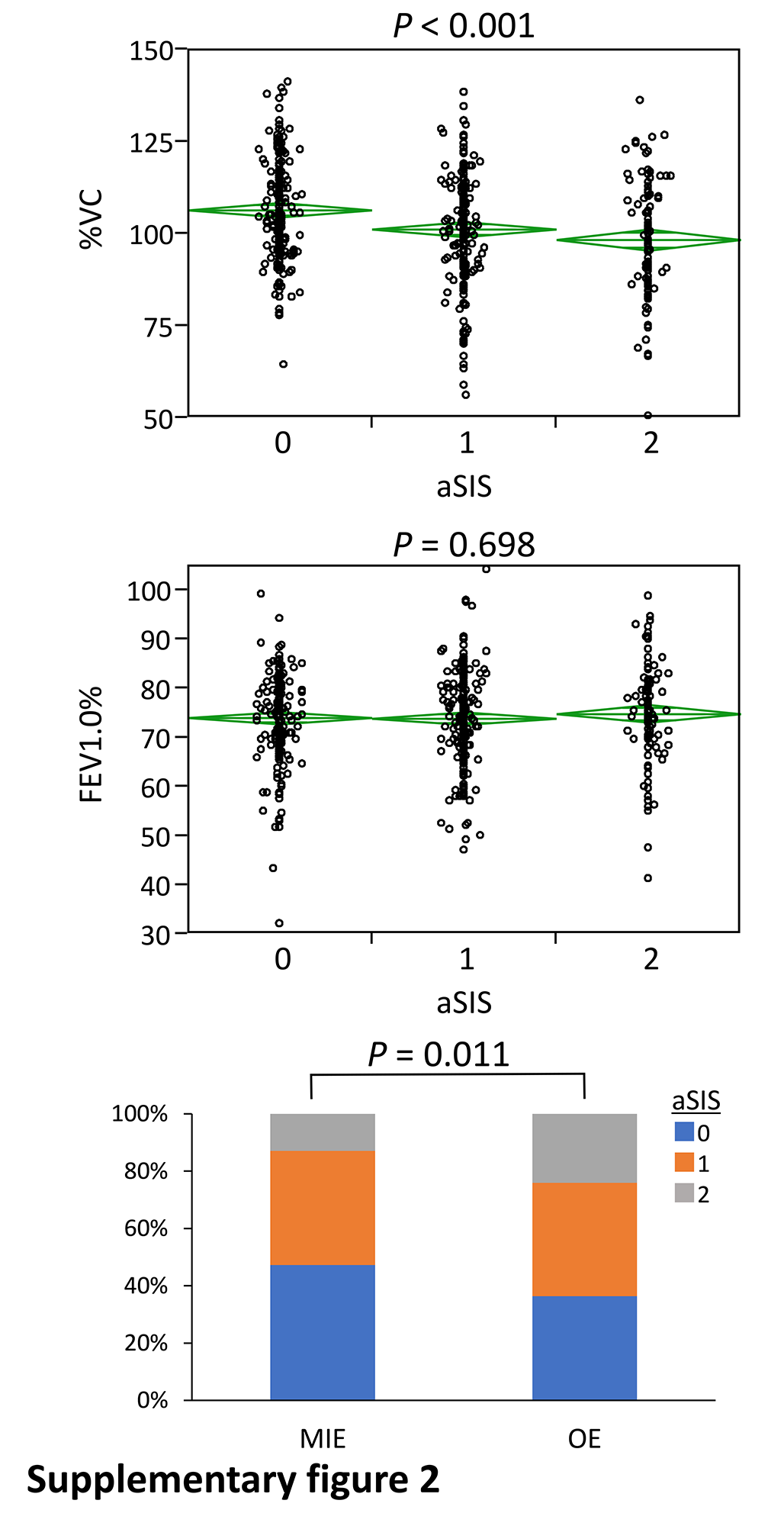

Supplement: Supplementary file 2 — Fig S2 [file AGS3-5-669-s004.tif]

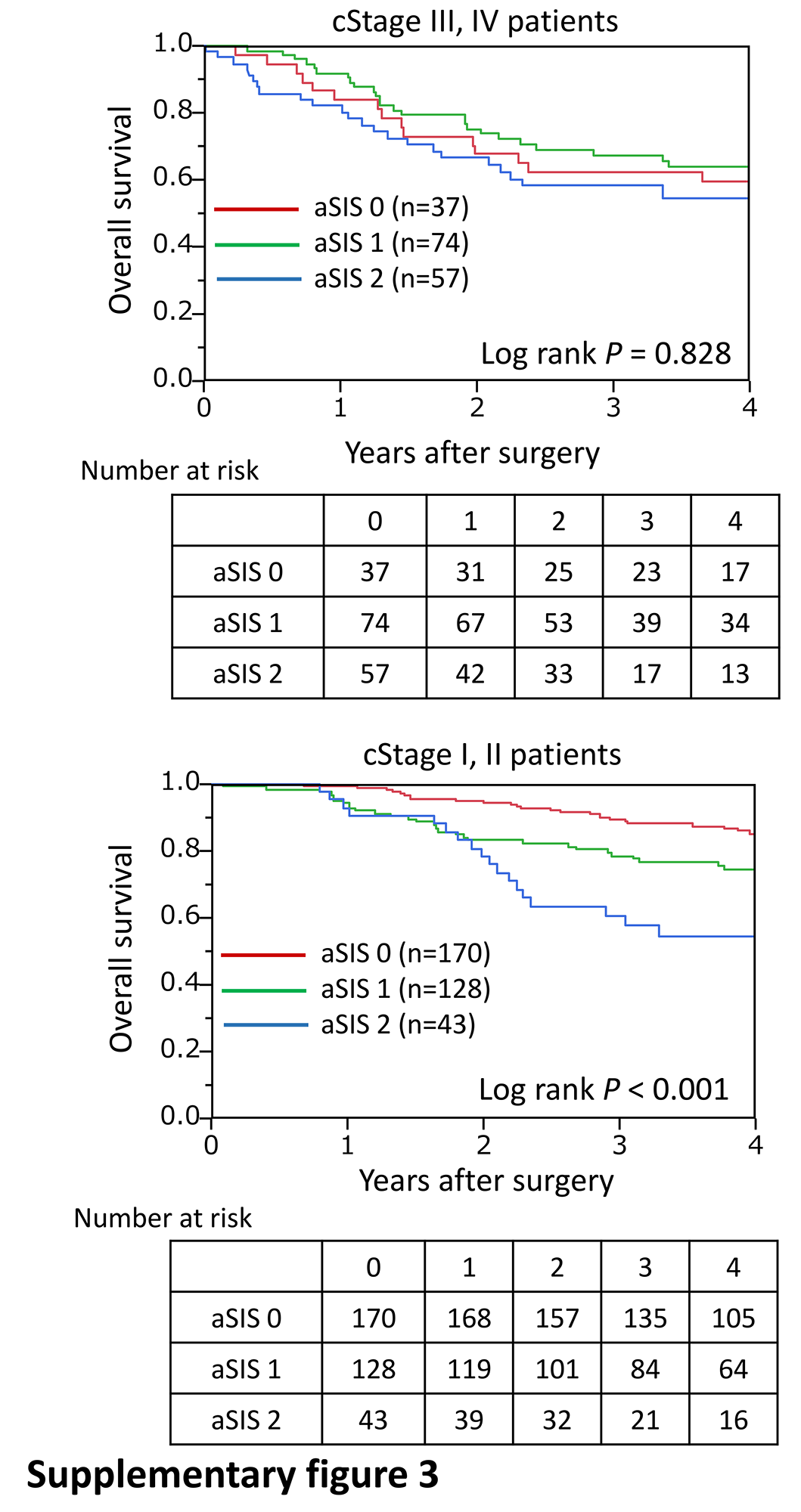

Supplement: Supplementary file 3 — Fig S3 [file AGS3-5-669-s002.tif]
