# Supplementary material for: Adapted systemic inflammation score as a novel prognostic marker for esophageal squamous cell carcinoma patients
Source: Ann Gastroenterol Surg. 2021 Jun 15;5(5):669–76. doi: 10.1002/ags3.12464 (PMC8452479; doi:10.1002/ags3.12464)
Supplement: Supplementary file 4 — Table S1‐4 [file AGS3-5-669-s001.docx]

**Supplementary table 1. Patients’ characteristics in preoperative treatment (-)**

| Variables |  | Total  N |  | aSIS | | | *P* value |
| --- | --- | --- | --- | --- | --- | --- | --- |
|  |  |  |  | 0 | 1 | 2 |  |
| All cases | 314 | | | 163 | 111 | 40 |  |
| Mean age ± SD | 66.6 ± 8.2 | | | 64.5 ± 8.2 | 69.3 ± 7.8 | 67.9 ± 9.3 | < 0.01 |
| Sex, Male | 277 (88%) | | | 143 (88%) | 96 (86%) | 38 (95%) | 0.28 |
| Performance status |  | | |  |  |  | < 0.01 |
| 0 | 277 (88%) | | | 152 (93%) | 96 (86%) | 29 (73%) |  |
| 1 | 31 (10%) | | | 11 (7%) | 11 (10%) | 9 (22%) |  |
| 2 | 6 (2%) | | | 0 (0%) | 4 (4%) | 2 (5%) |  |
| Body Mass Index |  | | |  |  |  | 0.048 |
| < 18.5 | 34 (11%) | | | 14 (9%) | 12 (11%) | 8 (20%) |  |
| 18.5 ≤, < 25 | 227 (72%) | | | 113 (69%) | 86 (77%) | 28 (70%) |  |
| 25 ≤ | 53 (17%) | | | 36 (22%) | 13 (12%) | 4 (10%) |  |
| Alcohol use, Yes | 297 (95%) | | | 157 (96%) | 104 (94%) | 36 (90%) | 0.28 |
| Tobacco use, Yes | 265 (84%) | | | 134 (82%) | 95 (86%) | 36 (90%) | 0.41 |
| Comorbidity, Present | 236 (75%) | | | 111 (68%) | 93 (84%) | 32 (80%) | < 0.01 |
| Tumor location |  | | |  |  |  | 0.87 |
| Ce | 1 (1%) | | | 1 (1%) | 0 (0%) | 0 (0%) |  |
| Ut | 44 (14%) | | | 25 (14%) | 16 (14%) | 3 (7%) |  |
| Mt | 167 (52%) | | | 87 (53%) | 59 (53%) | 21 (53%) |  |
| Lt | 94 (30%) | | | 46 (28%) | 33 (30%) | 15 (37%) |  |
| Ae | 8 (3%) | | | 4 (2%) | 3 (3%) | 1 (3%) |  |
| Clinical stage |  | | |  |  |  | < 0.01 |
| I | 252 (50%) | | | 140 (68%) | 89 (44%) | 23 (23%) |  |
| II | 89 (17%) | | | 30 (14%) | 39 (19%) | 20 (20%) |  |
| III | 143 (28%) | | | 26 (13%) | 68 (34%) | 49 (49%) |  |
| IV | 25 (5%) | | | 11 (5%) | 6 (3%) | 8 (8%) |  |
| Postoperative treatment |  | | |  |  |  | 0.44 |
| Present | 70 (22%) | | | 42 (26%) | 21 (19%) | 7 (17%) |  |
| Absent | 244 (78%) | | | 121 (74%) | 90 (81%) | 33 (83%) |  |

SD; Standard deviation

**Supplementary table 2. Patients’ characteristics in preoperative treatment (+)**

| Variables |  | Total  N |  | aSIS | | | *P* value |
| --- | --- | --- | --- | --- | --- | --- | --- |
|  |  |  |  | 0 | 1 | 2 |  |
| All cases | 195 | | | 44 | 91 | 60 |  |
| Mean age ± SD | 65.9 ± 7.5 | | | 64.9 ± 7.2 | 65.8 ± 7.5 | 66.7 ± 7.6 | 0.048 |
| Sex, Male | 169 (87%) | | | 35 (80%) | 80 (88%) | 54 (90%) | 0.29 |
| Performance status |  | | |  |  |  | 0.63 |
| 0 | 166 (85%) | | | 38 (86%) | 79 (87%) | 49 (82%) |  |
| 1 | 26 (13%) | | | 6 (14%) | 10 (11%) | 10 (17%) |  |
| 2 | 3 (2%) | | | 0 (0%) | 2 (2%) | 1 (1%) |  |
| Body Mass Index |  | | |  |  |  | 0.15 |
| < 18.5 | 27 (14%) | | | 2 (5%) | 14 (16%) | 11 (18%) |  |
| 18.5 ≤, < 25 | 141 (72%) | | | 33 (75%) | 65 (71%) | 43 (72%) |  |
| 25 ≤ | 27 (14%) | | | 9 (20%) | 12 (13%) | 6 (10%) |  |
| Alcohol use, Yes | 186 (95%) | | | 40 (91%) | 88 (97%) | 58 (97%) | 0.33 |
| Tobacco use, Yes | 171 (88%) | | | 34 (77%) | 81 (89%) | 56 (93%) | 0.042 |
| Comorbidity, Present | 156 (80%) | | | 34 (77%) | 73 (80%) | 49 (82%) | 0.86 |
| Tumor location |  | | |  |  |  | 0.70 |
| Ce | 1 (1%) | | | 0 (0%) | 1 (1%) | 0 (0%) |  |
| Ut | 28 (14%) | | | 6 (14%) | 16 (18%) | 6 (10%) |  |
| Mt | 112 (57%) | | | 25 (57%) | 54 (59%) | 33 (55%) |  |
| Lt | 49 (25%) | | | 12 (27%) | 18 (20%) | 19 (32%) |  |
| Ae | 5 (3%) | | | 1 (2%) | 2 (2%) | 2 (3%) |  |
| Clinical stage |  | | |  |  |  | 0.10 |
| I | 8 (4%) | | | 3 (7%) | 3 (3%) | 2 (3%) |  |
| II | 45 (23%) | | | 11 (25%) | 25 (27%) | 9 (15%) |  |
| III | 120 (62%) | | | 21 (48%) | 57 (63%) | 42 (70%) |  |
| IV | 22 (11%) | | | 9 (20%) | 6 (7%) | 7 (12%) |  |
| Postoperative treatment |  | | |  |  |  | 0.38 |
| Present | 26 (13%) | | | 8 (18%) | 9 (10%) | 9 (15%) |  |
| Absent | 169 (87%) | | | 36 (82%) | 82 (90%) | 51 (85%) |  |

SD; Standard deviation

**Supplementary table 3. Cox regression analysis for overall survival in preoperative treatment (-)**

| Variables | Univariate analysis | |  | Multivariate analysis | |
| --- | --- | --- | --- | --- | --- |
|  | HR (95% CI) | *P* value |  | HR (95% CI) | *P* value |
| Age (≥ 65 vs < 65) | 1.75 (1.11-2.80) | 0.014 |  | 1.50 (0.93-2.46) | 0.094 |
| Sex (male vs female) | 0.90 (0.50-1.81) | 0.75 |  |  |  |
| Brinkman Index (≥ 800 vs < 800) | 0.84 (0.53-1.30) | 0.43 |  |  |  |
| Body Mass Index (≥ 25 vs < 25) | 0.86 (0.44-1.53) | 0.62 |  |  |  |
| (< 18.5 vs ≥ 18.5) | 1.78 (0.93-3.13) | 0.077 |  |  |  |
| Performance Status (1, 2 vs 0) | 2.63 (1.51-4.34) | < 0.01 |  | 1.91 (1.07-3.27) | 0.030 |
| comorbidity (+ vs -) | 1.51 (0.90-2.66) | 0.12 |  |  |  |
| cStage (III, IV vs I, II) | 2.20 (1.13-3.92) | 0.023 |  | 1.71 (0.85-3.16) | 0.12 |
| Preoperative aSIS (1 vs 0) | 1.96 (1.21-3.20) | < 0.01 |  | 1.68 (1.02-2.77) | 0.041 |
| (2 vs 0) | 2.85 (1.52-5.15) | < 0.01 |  | 2.27 (1.18-4.19) | 0.015 |
| Postoperative complications  CDc ≥ IIIb (+ vs -) | 1.67 (0.86-2.97) | 0.13 |  |  |  |

HR; Hazard ratio, CI; Confidence interval, CDc; Clavien-Dindo classification

**Supplementary table 4. Cox regression analysis for overall survival in preoperative treatment (+)**

| Variables | Univariate analysis | |  | Multivariate analysis | |
| --- | --- | --- | --- | --- | --- |
|  | HR (95% CI) | *P* value |  | HR (95% CI) | *P* value |
| Age (≥ 65 vs < 65) | 1.38 (0.88-2.20) | 0.16 |  |  |  |
| Sex (male vs female) | 1.72 (0.85-4.11) | 0.14 |  |  |  |
| Brinkman Index (≥ 800 vs < 800) | 1.22 (0.53-1.27) | 0.37 |  |  |  |
| Body Mass Index (≥ 25 vs < 25) | 0.59 (0.26-1.14) | 0.12 |  |  |  |
| (< 18.5 vs ≥ 18.5) | 1.75 (0.93-3.07) | 0.082 |  |  |  |
| Performance Status (1, 2 vs 0) | 1.52 (0.85-2.56) | 0.15 |  |  |  |
| comorbidity (+ vs -) | 1.44 (0.83-2.67) | 0.20 |  |  |  |
| cStage (III, IV vs I, II) | 1.27 (0.78-2.15) | 0.35 |  |  |  |
| Preoperative aSIS (1 vs 0) | 0.99 (0.58-1.75) | 0.97 |  |  |  |
| (2 vs 0) | 1.23 (0.68-2.26) | 0.50 |  |  |  |
| Postoperative complications  CDc ≥ IIIb (+ vs -) | 1.67 (0.92-2.84) | 0.090 |  |  |  |

HR; Hazard ratio, CI; Confidence interval, CDc; Clavien-Dindo classification
