# Supplementary material for: Adapted systemic inflammation score as a novel prognostic marker for esophageal squamous cell carcinoma patients
Source: Ann Gastroenterol Surg. 2021 Jun 15;5(5):669–76. doi: 10.1002/ags3.12464 (PMC8452479; doi:10.1002/ags3.12464)
Supplement: Supplementary file 5 — Supplementary Material [file AGS3-5-669-s005.docx]

**Supplementary figure 1.** Histogram and Kaplan-Meier curves using X-tile software.

**Supplementary figure 2.** The colleration between aSIS and patients’ characteristics affecting pulmonary morbidity such as a respiratory function or surgical procedure.

**Supplementary figure 3.** Kaplan-Meier curves for OS in patients with esophageal squamous cell carcinoma, according to the aSIS stratification. The upper panel includes cStage III and IV patients. The lower panel includes cStage I and II patients.
